# Fossil-calibrated phylogenies of Southern cave wētā show dispersal and extinction confound biogeographic signal

**DOI:** 10.1098/rsos.231118

**Published:** 2024-02-14

**Authors:** Eddy J. Dowle, Steven A. Trewick, Mary Morgan-Richards

**Affiliations:** ^1^ Department of Anatomy, University of Otago, 9016 Dunedin, New Zealand; ^2^ Ecology, School of Natural Sciences, Massey University Manawatū, Private Bag 11 222, Palmerston North 4410, New Zealand

**Keywords:** cave crickets, dispersal, extinction, mitogenome, molecular clock, subantarctic

## Abstract

The biota of continents and islands are commonly considered to have a source–sink relationship, but small islands can harbour distinctive taxa. The distribution of four monotypic genera of Orthoptera on young subantarctic islands indicates a role for long-distance dispersal and extinction. Phylogenetic relationships were inferred from whole mtDNA genomes and nuclear sequences (45S cassette; four histones). We used a fossil and one palaeogeographic event to calibrate molecular clock analysis. We confirm that neither the Australian nor Aotearoa-New Zealand Rhaphidophoridae faunas are monophyletic. The radiation of Macropathinae may have begun in the late Jurassic, but trans-oceanic dispersal is required to explain the current distribution of some lineages within this subfamily. Dating the most recent common ancestor of seven island endemic species with their nearest mainland relative suggests that each existed long before their island home was available. Time estimates from our fossil-calibrated molecular clock analysis suggest several lineages have not been detected on mainland New Zealand, Australia, or elsewhere most probably due to their extinction, providing evidence that patterns of extinction, which are not consistently linked to range size or lineage age, confound biogeographic signal.

## 1. Introduction

The current geographical distributions of extant species often form the basis of taxonomic and biogeographic hypotheses. For example, terrestrial taxa restricted to the Southern Hemisphere have been used to infer Gondwanan origin and affinities of many taxa [1]. However, the suitability of current distributions to reconstruct evolutionary history varies [2] as the approach rests upon the assumption that current ranges represent past distributions. In particular, the role of extinction in determining observable biogeographic patterns is too often ignored [3]. Rates of extinction are likely to vary in time and space, for example rapid climate change is likely to increase rates of extinction and the restricted land area of islands is likely to elevate rates of extinction of terrestrial organisms compared with continents [4]. The biological significance of islands as evolutionary engines of diversification is well recognized [4–8], yet biogeographers often focus on continents when considering the long-term development of terrestrial biotas. The implicit assumption that continental environments are likely to have provided more persistent opportunities seems broadly reasonable for some types of organism at least [9] although even large landmasses are known to be subject to profound shifts of environmental conditions and biological distributions [10]. Oceanic islands can emerge abruptly providing novel opportunities for colonizing organisms [11] but may be of short duration in geological terms [12–14]. This inevitably means that continental representatives of a biological group tend to reflect older divergence events compared with those detected within island taxa, and this is broadly borne out in some taxa (e.g. amphibians [15]), but not others (cryptogams, [16]; flycatchers, [17,18]). Richly sampled lineages and robust data-rich phylogenies with independent time calibration can test alternative predictions about the parent–daughter relationships of taxa even where spatial pattern alone remains ambiguous [19].

**Figure 1.**
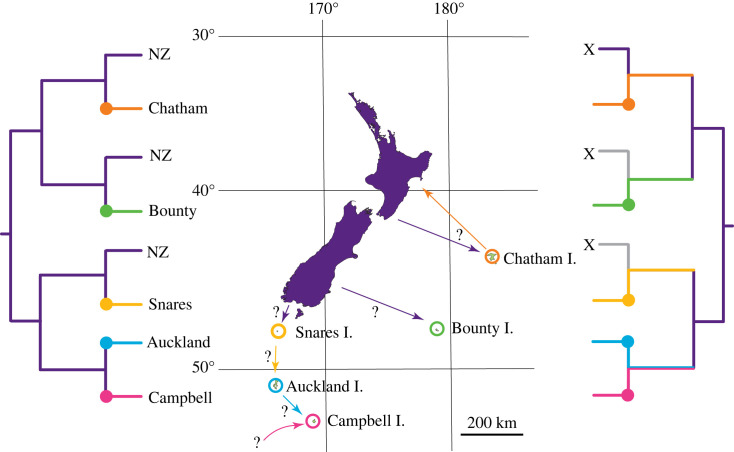
The geographical range of the Macropathinae of Aotearoa-New Zealand includes mainland (NZ) and offshore islands hosting four endemic monotypic genera. Two contrasting hypotheses are illustrated. Left: Islands are home to recent arrivals with relatives on mainland New Zealand sharing a common ancestor no older than the age of the island land surface. Right: Islands harbour lineages without close relatives due to extinction or failure to sample (X) of sister lineages on mainland New Zealand. Coloured spots on tree indicate earliest existence of each island. Arrows indicate potential dispersal from source to current range with ? indicating unknown time and direction.

When islands are sinks for a subset of diversity from larger landmasses, we might expect lineage age on islands to be concordant with the age of the land surface (figure 1); however, stem age is not a good proxy for time of island colonization [20]. If the most recent common ancestor of an island endemic and its sister on the mainland existed before the island existed, a parsimonious inference is the existence of a former mainland population from which individuals were derived before taking up residence on the island. When the mainland descendants of the common ancestor are unavailable, due to extinction or a failure to sample, island calibrations can mislead estimates of divergence times [21]. This results from the universal phenomenon in phylogenetic inference that branch lengths increase as terminal taxa are removed, with the penultimate situation being a singleton ‘relict' extant taxon representing a formerly diverse clade [22]. Extinction (or failure to sample) will then remove the association between island age and phylogenetic estimates of endemic fauna and flora (figure 1).

The insect family Rhaphidophoridae (Orthoptera, Ensifera) comprises nocturnal crickets variously known as cave crickets or camel crickets, or in New Zealand cave wētā or tokoriro, and has a global distribution with 867 recognized species [23]. Phylogenetic relationships within this family suggest the subfamilies form clades that have distinct geographical distributions [24]. Thus, species within the subfamilies Dolichopodainae and Troglophilinae are found only in the Mediterranean Region, Rhaphidophorinae is in southeast Asia and Ceuthophilinae representatives are restricted to North America. This geographical distribution might be explained by continental drift vicariance if the ancestral Rhaphidophoridae existed on Pangaea; however, spatial relationships among taxa in the Southern Hemisphere are more complex [25] despite oceanic isolation of landmasses since break-up of Gondwana. Within Rhaphidophoridae, the subfamily Macropathinae Karny, 1930 is a monophyletic clade sister to the southeast Asian Aemodogryllinae and Rhaphidophorinae [24]. Species within the Macropathinae clade are present on continental South Africa, South America, Australia, Aotearoa-New Zealand (where they have the highest species diversity) and on many oceanic islands. This distribution suggests that transoceanic dispersal has been an important process determining their evolutionary history, extant diversity and current distribution.

Previous phylogenetic analyses of Rhaphidophoridae have used geological calibrations to estimate time of most recent common ancestor of the group [24,26]. This approach has severe limitations for biogeographic analysis as calibrations depend on assumptions about the role of continental drift in the distribution of taxa, which is often the subject of the question being considered. Interestingly, many species of Rhaphidophoridae are restricted to islands. For example, Crete is inhabited by *Dolichopoda paraskevi* (Aegean Sea, Greece), *Parudenus falklandicus* lives on the Falkland Islands in the South Atlantic Ocean and *Notoplectron campbellense* on subantarctic Campbell Island in the Southern Ocean. Many oceanic islands are geologically relatively young and have never had direct contact with other land, having formed from volcanic activity in the last 10 Myr [13]. The occurrence of Rhaphidophoridae on southern oceanic islands suggests that at least some southern cave wētā owe their current distribution to chance dispersal across oceans, so there is potential to improve understanding of the timing of the Macropathinae radiation by incorporating fossil calibrations in appropriate molecular phylogenetic analysis. The current distribution of taxa and their island homes could provide a signature of dispersal and extinction.

In New Zealand, 63 species of Rhaphidophoridae are recognized and another dozen or more await formal description (electronic supplementary material, table S1); all within the subfamily Macropathinae. The fauna of Aotearoa-New Zealand include six monotypic genera, five of which are restricted to offshore island groups; *Ischyroplectron* on Bounty Island, *Insulanoplectron* on Snares Island, *Dendroplectron* on Auckland Island, *Novoplectron* on Chatham Islands and *Paraneonetus* on Manawatāwhi/Three Kings Islands. On subantarctic Campbell Island is the endemic species *Notoplectron campbellense* which was considered the only member of this genus until 2022 when the New Zealand alpine species *Pharmacus brewsterensis* became *Notoplectron brewsterense* [27]. The islands that are home to these diverse Rhaphidophoridae are between 8 and 670 km from mainland New Zealand (figure 1), and none have had terrestial contact with other land. Interestingly, the surface of two of these islands has been ice-free for only about 20 thousand years (Campbell and Auckland Islands [28]) and therefore all their terrestrial fauna must have arrived recently (figure 1). Similarly, the Chatham Island land surfaces are also relatively young, having risen above the ocean within the last 2–4 Myr [29,30]. Therefore, the insects living on Chatham, Campbell and Auckland Islands must have ancestors that dispersed across the Southern Ocean relatively recently (Pliocene/Pleistocene). These island endemics (including four monotypic genera) either have living relatives on some other land masses that have remained unrecognized by taxonomy, or their ancestral populations have never been sampled due to lack of resources or their extinction. Extinction is a key component of evolution but one that can readily mislead biogeographic inference [3,22].

We used high-throughput sequencing of whole-genomic DNA to assemble entire mitochondrial genomes and multi-copy nuclear markers for molecular phylogenetics. Our sampling of all except two endemic genera of Rhaphidophoridae from the New Zealand region and representatives from South America, South Africa and Australia allow us to determine evolutionary relationships among the New Zealand fauna. Explicitly, we tested the hypothesis that members of the genus *Macropathus* are not part of a New Zealand monophyletic clade, despite being endemic to New Zealand, and that the Australian taxa are not monophyletic. We also tested the suggestion that the New Zealand diversity arose during the Neogene (within the last 25 Myr; [24]). Using molecular calibrations from both fossils and geology (age of land surface of Chatham Islands), we infer time since last common ancestor of island endemics. If the common ancestor is older than the land surface, we can infer failure to sample [21].

## 2. Material and methods

### 2.1. Taxa and sampling

**Figure 2.**
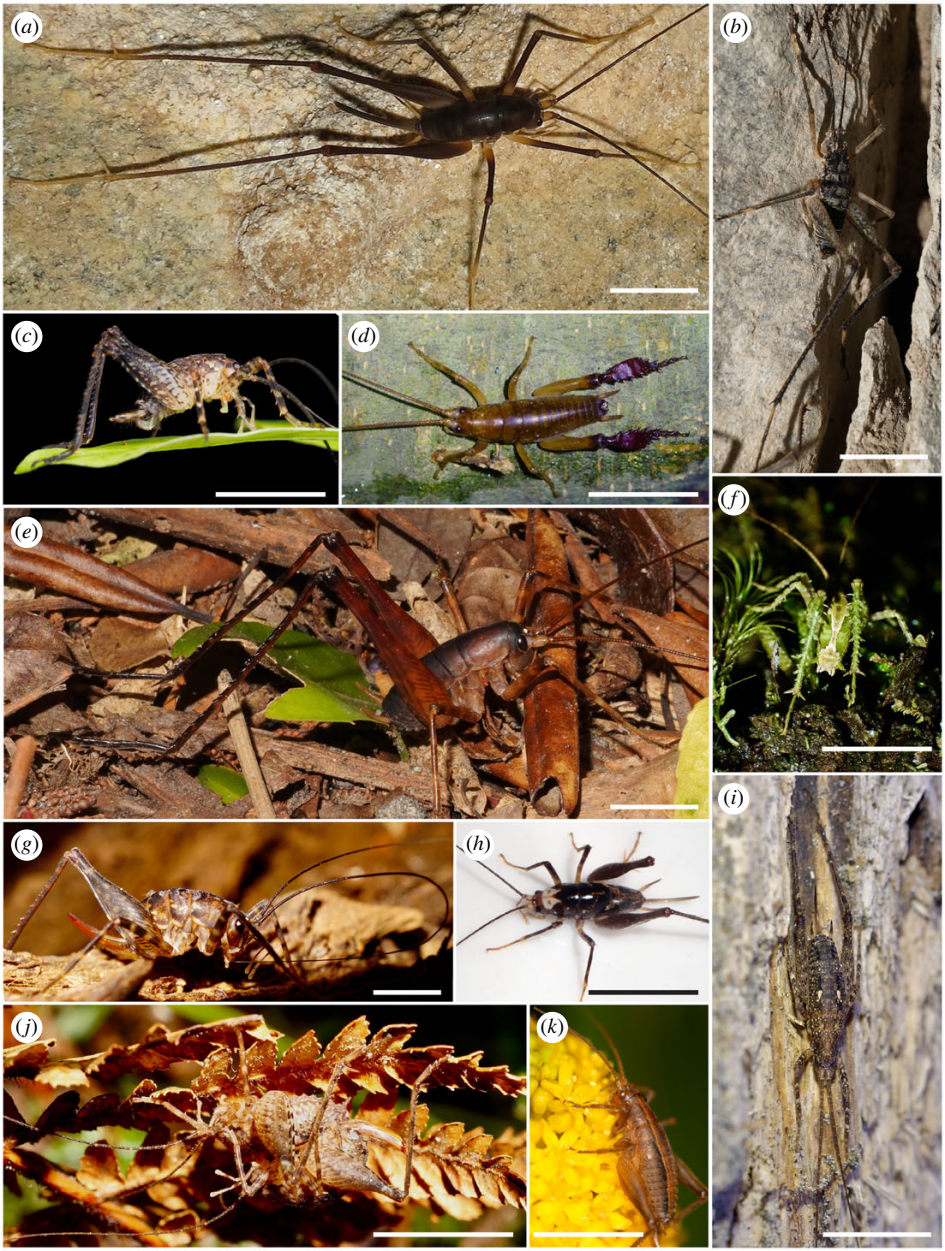
Images of Rhaphidophoridae species endemic to Aoteoroa-New Zealand illustrating their morphological diversity. Scale bars are approximately 10 mm. (*a*) *Macropathus filifer* (photo credit Alice Shanks); (*b*) *Petrotettix serratus* (photo credit Steve Trewick); (*c*) *Neonetus* n. Sp-1. (photo credit Emily Roberts); (*d*) *Talitropsis megatibia* (photo credit Christine Painting); (*e*) *Pachyrhamma longicaudum* (photo credit Steve Trewick); (*f*) *Maotoweta virescens* (photo credit Dave Holland); (*g*) *Miotopus diversus* (photo credit Steve Trewick); (*h*) New Genus-1 (photo credit Steve Trewick); (*i*) *Isoplectron armatum* (photo credit Christopher Stephens); (*j*) *Pleioplectron hudsoni* (photo credit Steve Trewick); (*k*) *Notoplectron campbellense* (photo credit Alex Fergus).

The New Zealand Rhaphidophoridae are morphologically and ecologically diverse (figure 2). Our specimens were collected from caves, forests, high alpine scree, subantarctic scrub and urban environments (table 1). Previous taxonomy placed the six species of *Talitropsis* in the tribe Talitropsini Gorochov, 1988 and all other New Zealand species within the tribe Macropathini Karny, 1930. However, molecular phylogenetic analysis suggests that rather than *Talitropsis* being distinct, we could recognize the genus *Macropathus* as distinct due to its placement as sister to a clade that includes New Zealand and South American species of Rhaphidophoridae [24,26]. Thus, we include two species from Chile, two species from Tasmania (representing two distinct clades; [26]), and one each from South Africa and North America (table 1). We endeavoured to include the full scope of phylogenetic diversity in New Zealand by sampling one, two or three species from 16 of the 18 described New Zealand genera plus undescribed species from two unnamed genera (table 1). One genus not sampled, *Setascutum,* is almost certainly a synonym of *Isoplectron* [31], the other, *Paraneonetus*, is a monotypic island endemic from Three Kings/Manawatawhi. We included seven species endemic to five oceanic islands in the New Zealand region. Within New Zealand, authority to collect was provided by the New Zealand Department of Conservation (permit numbers: WE/145/RES; WE/264/RES; 37024-FAU; TW-32116-FAU; TT-15419-FAU; ECHB-15515-RES; WE/31465/FAU; WA-22197-RES; CA-17825-FAU; CA-15142-FAU; NM-15823-RES; NM-32444-FAU; 11/592; OT-19868-RES; SO-19085-FAU; 47966-FAU). Within Chile, authority to collect was provided by La Corporación Nacional Forestal (number 09/2011). Additional material was provided from Sapienza University of Rome; Te Papa Tongarewa Museum of New Zealand; NIWA Taihoro Nukurangi; Department of Conservation Te Papa Atawhia. Year and collector information is provided with authority and link to photograph of the specimen (via *i*Naturalist) where available (electronic supplementary material, table S2).

**Table 1.** Rhaphidophoridae specimens used to assemble whole mitochondrial genome sequences and nuclear 45S cassette and histone DNA sequences for phylogenetic analysis, and the outgroup taxa included in this study. MtDNA assembly includes putative control regions excluded from subsequent analysis. MPN code refers to material held in Massey University Manawatū Museum collection. NI, New Zealand North Island; SI, New Zealand South Island.

MPN code	species	subfamily	region	location	mtDNA (bp)	Nuclear DNA (bp)	GenBank accessions (mtDNA; 28S; 18S)
CW1010	*Pachyrhamma longipes*	Macropathinae	New Zealand	forest, Hawkes Bay, NI	15 871	6377	OR551717; OR520208; OR520247
CW1657	*Pachyrhamma edwardsii*	Macropathinae	New Zealand	forest, Kapiti Island	15 618	6377	OR551719; OR520210; OR520249
CW3260	*Pachyrhamma edwardsii*	Macropathinae	New Zealand	forest, Gouland Downs, SI	15 462	6377	OR551729; OR520216; OR520260
CW1970	*Miotopus diversus*	Macropathinae	New Zealand	forest, Waioeka Gorge, NI	14 89	6377	OR551723; OR520222; OR520253
CW3	*Novoplectron serratum*	Macropathinae	Chatham Rise	forest, Chatham Islands	15 745	6377	OR551709; OR520207; OR520237
TP23	*Ischyroplectron isolatum*	Macropathinae	Subantarctic Ocean	scrub, Bounty Islands	15 898	6377	OR551735; OR520226; OR520267
CW2680	*Pleioplectron hudsoni*	Macropathinae	New Zealand	forest, Kaweka Range, NI	15 787	6377	OR551726; OR520213; OR520256
CW3383	*Pleioplectron rodmorrisi*	Macropathinae	New Zealand	alpine, Seaward Kaikouras, SI	15 844	6377	OR551730; OR520218; OR520262
CWDEN	New Genus 1	Macropathinae	New Zealand	forest, West Coast, SI	15 354	6377	OR520220; OR520265
CW1335	*Insulanoplectron spinosum*	Macropathinae	Subantarctic Ocean	scrub, Snares I	15 883	6377	OR551718; OR520209; OR520248
CW120	*Neonetus* n. sp-1	Macropathinae	New Zealand	forest, Hawkes Bay, NI	15 322	6377	OR551710; OR520227; OR520239
CW1776	*Neonetus* n. sp-1	Macropathinae	New Zealand	forest, Rotorua, NI	16 281	6377	OR551720; OR520231; OR520250
CW1870	*Pallidoplectron* n. sp.	Macropathinae	New Zealand	forest, Hawkes Bay, NI	15 154	6377	OR551722; OR520233; OR520252
CW285	*Pharmacus senex*	Macropathinae	New Zealand	alpine, Old Man Range, SI	15 251	6377	OR551713; OR520229; OR520243
CW3245	*Petrotettix serratus*	Macropathinae	New Zealand	alpine, Seaward Kaikouras, SI	15 775	6377	OR551728; OR520234; OR520259
CW325	*Isoplectron armatum*	Macropathinae	New Zealand	forest, Canterbury, SI	15 326	6377	OR551714; OR520230; OR520244
CW1830	*Talitropsis sedilloti*	Macropathinae	New Zealand	forest, Hawkes Bay, NI	16 057	6377	OR551721; OR520232; OR520251
CW212	*Talitropsis crassicruris*	Macropathinae	Chatham Rise	forest, Chatham I	15 106	6377	OR551711; OR520225; OR520240
CW219	*Talitropsis megatibia*	Macropathinae	Chatham Rise	woolshed, Chatham Islands	15 121	6377	OR551712; OR520228; OR520241
CW2153	*Notoplectron campbellense*	Macropathinae	Subantarctic Ocean	scrub, Campbell I	15 863	6377	OR551724; OR520211; OR520254
CW2625	*Notoplectron brewsterense*	Macropathinae	New Zealand	alpine, Fiordland, SI	15 978	6377	OR551725; OR520212; OR520255
CW2827	*Maotoweta virescens*	Macropathinae	New Zealand	forest, West Coast, SI	15 884	6377	OR551727; OR520215; OR520258
CW3369	New Genus 2	Macropathinae	New Zealand	forest, Rakiura Stewart I	15 655	6377	OR520217; OR520261
CW2709	*Dendroplectron aucklandense*	Macropathinae	Subantarctic Ocean	scrub, Auckland Islands	15 241	6377	OR551736; OR520214; OR520257
CW109	*Macropathus* sp.	Macropathinae	New Zealand	cave, Hawkes Bay, NI	15 981	6377	OR520204; OR520238
CW226B	*Macropathus filifer*	Macropathinae	New Zealand	cave, West Coast, SI	15 867	6377	OR520205; OR520242
CW5229	*Heteromallus spina*	Macropathinae	Chile	forest, Parque On Col	15 392	6377	OR551733; OR520221; OR520266
CW5232	*Heteromallus* sp.1	Macropathinae	Chile	forest, Parque On Col	15 147	6377	OR551734; OR520219; OR520264
CW725	*Micropathus cavernicola*	Macropathinae	Tasmania	cave, Marakoopa	14 945	6377	OR551715; OR520223; OR520245
CW736	*Parvotettix domesticus*	Macropathinae	Tasmania	Taronga, Hobart	15 861	6377	OR551716; OR520224; OR520246
CW3801	*Spelaeiacris monslamiensis*	Macropathinae	South Africa	cave, Hex River Mountain	16 399	6377	OR551731; OR520206; OR520236
	*Diestramima* sp.	Aemodogryllinae	Asia		16 346		KX057718
	*Diestramima tibetensis*	Aemodogryllinae	Asia		16 060		KX057740
	*Diestramima asynamora*	Aemodogryllinae	Asia		15 309		KX057726
	*Troglophilus neglectus*	Troglophilinae	Italy	cave	15 810		NC011306
CW4347	*Ceuthophilus*	Ceuthophilinae	USA	Moab Desert	15 727	6377	OR551732; OR520235; OR520263
	**outgroup**	**family**					
	*Henicus brevimucronatus*	Anostostomatidae	South Africa		15 140		NC028063
	*Pteranabropsis carli*	Anostostomatidae	China	forest	15 932		NC035420
	*Pteranabropsis carnarius*	Anostostomatidae	Vietnam	forest	16 119		NC035552
	*Pteranabropsis crenatis*	Anostostomatidae	China	forest	16 099		NC035553
	*Apteranabropsis* sp.	Anostostomatidae	Asia		16 027		KY364002
	*Tarragoilus diuturnus*	Prophalangopsidae	China		16 144		JQ999995
	*Cyphoderris monstrosa*	Prophalangopsidae	North America		16 590		NC028059
	*Stenopelmatus fuscus*	Stenopelmatoidea	North/Central America		15 767		NC028058
	*Conocephalus melaenus*	Tettigonioidea	China/Asia		15 852		KX057725
	*Lipotactes tripyrga*	Tettigonioidea	China		15 949		KX057736
	*Ruspolia dubia*	Tettigonioidea	China		14 971		NC009876

Individual specimens were identified using a combination of apical spines, characteristic male and female terminalia and body dimensions with reference to [27,32–38]. Specimens were stored in 98% ethanol before DNA extraction. For outgroup and molecular clock analysis, we downloaded DNA sequences from 11 orthopteran species from GenBank representing the families Tettigonioidea, Stenopelmatodidae, Anostostomatidae, Prophalangopsidae (table 1).

### 2.2. DNA sequencing

Each eukaryote cell has multiple mitochondria, so it is possible to assemble the whole mtDNA genome using short anonymous DNA sequences generated from high-throughput next-generation sequencing (NGS) approaches. Similarly, highly replicated markers such as the nuclear 45S ribosomal (rRNA) cassette and histones can also be assembled from the same skim sequencing data [2,39,40]. Despite biparental inheritance the 45S ribosomal cassette tends to be homogenized via concerted evolution [41]. The rDNA regions of the cassette are highly conserved and so show a slow rate of nucleotide substitution that has been used to study deep phylogenetic relationships [39,42,43]. By contrast, the ITS regions are not functionally constrained in the same way, and so have high substitution rates. A second nuclear gene family assembled here is the histone proteins H2A, H2B, H3 and H4 that are adjacent to one another in these Orthoptera.

Insect DNA was extracted using a high salt method [44,45] and quantified using Qubit fluorometry (Life Technologies, Thermo Fisher Scientific Inc.). Genomic DNA samples were paired-end sequenced with high-throughput sequencing on an Illumina HiSeq 2500 (either BGI or Macrogen) following fragmentation and indexing using the Illumina TruSeq Nano DNA kit. Resulting 100 bp or 150 bp paired-end reads were demultiplexed, and used to assemble DNA sequences for the whole mitochondrial genome, and where feasible the full nuclear 45S ribosomal (rRNA) cassette and set of four histones using Geneious v. 9.1.4 [46].

Mitochondrial genomes were obtained from each specimen using an iterative reference mapping approach. The first of the Macropathinae genome assemblies used an annotated mtDNA genome of a Rhaphidophoridae for initial mapping. Paired reads were iteratively mapped to the reference sequence in Geneious generating a novel consensus sequence, which was then used as a reference to remap the raw sequence reads. This process was repeated until all alignment gaps were filled by extension with the new sequence data and ambiguities resolved. Henceforth subsequent assemblies began with the more similar reference templates from our first New Zealand Macropathinae mtDNA genome. This approach has proved fast and efficient for grasshoppers [39]. Sequences were uploaded as raw fasta files to Mitos [47] for initial identification of protein coding regions, rDNAs and tRNAs. Annotations were transferred and individually cross-checked by comparison of reading frames, amino acid translation and RNA structure. Due to tRNA rearrangments, tRNA structure was rechecked using Arwen [48]. These sequence data have been submitted to the GenBank database (table 1).

We used the same approach to assemble, align and edit the nuclear loci 45S ribosomal cassettes and histone genes H2A, H2B, H3 and H4 for all Macropathinae. To seed this process, we used published orthopteran 5.8S rRNA sequences that are highly conserved among taxa to iteratively build the full 45S cassette. For histones, we used available partial H3 sequences to start the process and found by iterative mapping that these genes occupy the same chromosome in the Macropathinae.

### 2.3. Alignment and phylogenetic analysis

Alignments for each of the 13 coding, two rRNA and 22 tRNA genes were generated in Geneious Prime. Protein coding genes were aligned using the translational alignment function with the Mafft aligner (auto algorithm and invertebrate genetic code), the rRNA loci were aligned using the Mafft aligner (E-INS-i algorithm), while the tRNAs were aligned separately using the Mafft aligner (auto algorithm) before they were concatenated (16 mitogenome loci in total).

Two DNA sequence alignments were created for phylogenetic analysis. One dataset consisted of 47 taxa (36 Rhaphidophoridae and 11 outgroup taxa from four other orthopteran families; table 1). This full mitochondrial dataset was used for molecular clock analysis (see below). Another dataset consisted of 31 Rhaphidophoridae for which we had both mitochondrial and nuclear DNA sequences (table 1) allowing us to examine phylogenetic evidence from these independent genomes. We retained all of the 13 mitochondrial protein coding genes for this subset of taxa. Partial 18S and 28S were extracted from the full 45S cassette and concatenated with four histone exons for each sample prior to alignment. Phylogenetic relationships within the sampled 31 Rhaphidophoridae were inferred using a bootstrapped maximum-likelihood (ML) phylogeny estimated using IQ-Tree 2.2.2.2 [49]. A best-fit substitution model for each locus was estimated using ModelFinder [50] within IQ-Tree, and node support was assessed with 1000 ultrafast bootstrap replicates [51]. Full DNA alignments are available on Dryad [52].

### 2.4. Molecular clock analysis

We inferred the timing of the New Zealand cave wētā radiation using Beast2 v. 2.7.1 [53]. To calibrate the whole mtDNA phylogeny, we included taxa that represented a lineage suitable for the use of an Ensifera fossil calibration. As molecular clock analyses are sensitive to choice and placement of calibrations [54,55], the employment of several different fossils is preferred because it enables internal rate verification and exploration of prior assumptions. However, after exploring a number of additional fossil constraints within our analyses, convergence was possible with only one combination. We used one fossil calibration outside of the Rhaphidophoridae clade and one recent geological constraint within the New Zealand Macropathinae radiation:

1. Prophalangopsidae is a family of Ensifera with fossils from the Jurassic (201−145 Ma; [56,57], including three species of *Aboilus* fossils from the lower Jurassic period [58]. We constrained our molecular clock analysis so that the most recent common ancestor between the Prophalangopsidae (represented by *Tarragoilus diuturnus* and *Cyphoderris monstrosa*) and Anostostomatida + Stenopelmatidae existed approximately 160 Ma. Based on the fossil age and the effective sample size (ESS) values returned from the prior runs this calibration point was set in Beast with a gamma distribution with an offset of 157.3.
2. Crown age of endemic island lineages provide the opportunity to date *in situ* diversification and such ages represent a more reliable estimate of time of colonization than stem age [20]. Both species of *Talitropsis* cave wētā endemic to the Chatham Islands were sampled for this analysis. Given that Chatham Islands have been emergent for only the last 2­–4 Myr [29,30], we can be confident that the two endemic *Talitropsis* species must share a common ancestor within this time frame. Therefore, the biogeographic calibration point of the most recent common ancestor of *Talitropsis crassicruris* and *Talitropsis megatibia* was constrained in Beast using a normal distribution with a 1.0 offset (2.5% quantile −0.9, 97.5% quantile 2.96).

We used BEAUti2 v. 2.7.1 [53] to generate a Beast .xml file. A suite of divergence dating analyses were used to explore the data and compatibility of calibrations, before a final set-up was selected. Sixteen gene regions were analysed (13 coding genes, two rRNA genes and the concatenated tRNA genes). Protein coding genes were separated into first, second and third coding regions and the Clock and Tree models were linked across all genes. A Beast model test [59] was run on the five gene partitions (16S and 12S, tRNA, codon position 1, 2 and 3), while a Fast Relaxed Clock Log Normal and a Calibrated Yule Model (restricted) were used for the clock and tree prior respectively. Two Beast runs were done, initially changing the parameters as suggested, before four final chains were run to confirm convergence. Final runs were sampled every 1000 generations from a total of 200 million generations. Convergence was determined using Tracer, before logs and trees were combined using LogCombiner.

Time estimated phylogenies were obtained using Beast2 analyses. Tracer was used to investigate the Bayesian outputs, and ESS statistics (prior, posterior, tree likelihood, tree height) were used to indicate whether the posterior space of the models was sufficiently explored. ESS values greater than 200 are considered sufficient for the analyses to be informative [60,61]. The four chains were checked for comparable convergence in Tracer before being combined used LogCombiner (resampling down 43 K trees) and Maximum clade credibility trees with median heights were generated in TreeAnnotator v. 2.4.4 [53] and edited in FigTree, where estimated dates of divergence could be visualized. We used 95% highest posterior density (HPD) intervals to represent the uncertainty of inferred divergence times [62]. New Zealand eScience Infrastructure (NeSI) was used for all phylogenetic tree construction and divergence dating analyses.

## 3. Results

Complete mtDNA genome sequences were assembled for 32 Rhaphidophoridae individuals (table 1), all comprising the expected arrangement of 13 protein coding genes, 22 tRNAs, two rDNAs and a putative control region (electronic supplementary material, table S3). The length of the 13 concatenated protein coding genes varied from 186 bp (ATP8) to 1731 bp (ND5). The orientation and order of genes was identical to that of other Rhaphidophoridae with the exception that the two *Macropathus* species studied had several tandem repeats of DNA sequence (98 bp) that included tRNA-Ser2 between the cob and nad1 (electronic supplementary material, file, mitochondrial gene arrangment).

Nuclear 45S rRNA cassettes and histone genes, H3 and H4, were assembled for 31 Rhaphidophoridae specimens. The resulting alignment showed high sequence conservation at 28S, 18S, 5.8S, H3 and H4, contrasting with higher sequence variation at ITS1, ITS2 and the intergenic spacer between H3 and H4 including many Insertion–deletion mutations (INDELs).

**Figure 3.**
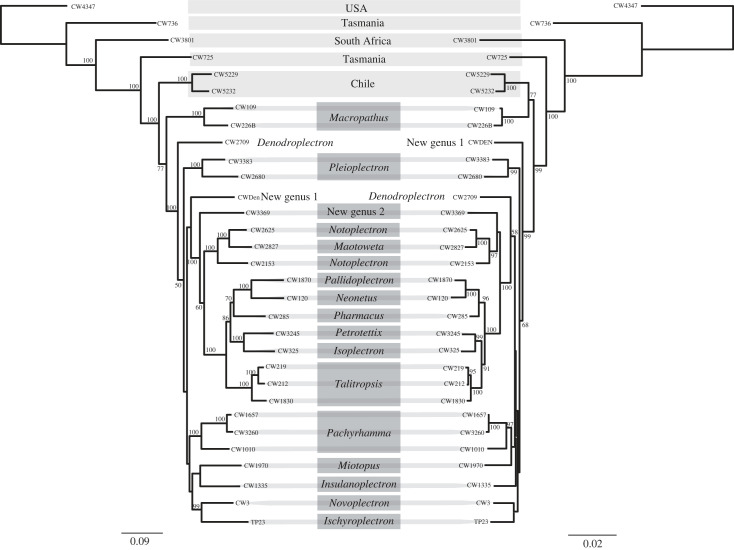
Concordance of phylogenetic relationships within the New Zealand (dark grey) radiation of southern cave crickets/wētā (Rhaphidophoridae; subfamily Macropathine) inferred from mitochondrial and nuclear DNA sequence. Left: ML tree from alignment of mitochondrial protein coding gene sequences (11 205 bp); Right: ML tree from alignment of nuclear DNA sequences from partial 18S and 28S (4907 bp) and four histones (1470 bp).

**Figure 4.**
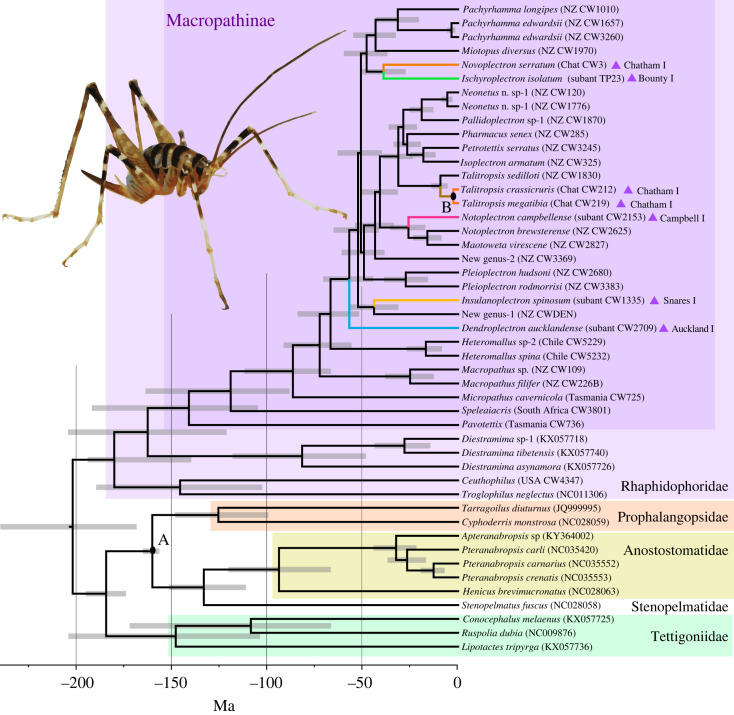
Phylogenetic hypothesis of Rhaphidophoridae from analysis of an alignment of whole mitochondrial DNA sequences using ML. Time calibration used Jurassic fossils at the node representing the common ancestor of the Prophalangopsidae and their sister families (Anostostomatidae and Stenopelmatidae; Node A), and the age of the land surfaces of the Chatham Islands constrained the common ancestor of *Talitropsis crassicruris* and *Talitropsis megatibia* (Node B). Lineages endemic to oceanic islands are coloured. Inset shows adult female *Pachyrhamma edwardsii*.

Comparison of the nuclear and mitochondrial markers using a phylogenetic analysis of 11 205 bp of mtDNA and 6377 bp nuclear sequence, show concordance (figure 3). In neither the phylogenies inferred from the mtDNA sequence nor from the nuclear DNA sequence were the two Tasmanian taxa sisters (figure 3). The evolutionary relationship of the two species from Chile with respect to the New Zealand diversity is unresolved due to variation among analyses (figures 3 and 4). The species from Chile (*Hetermallus* spp.) are either sister to or nested within the New Zealand phylogenetic diversity, with the tree inferred from nuclear data and the larger mtDNA dataset placing this South American lineage within the New Zealand clade (figures 3 and 4). Relationships among the New Zealand species inferred using the two datasets were very similar. Where we had more than one representative of a genus the specimens formed well-supported clades (e.g. *Pachyrhamma, Talitropsis, Pleioplectron, Macropathus*).

In our analysis with 47 taxa, the Northern Hemisphere Rhaphidophoridae lineages (represented by *Troglophilus* and *Ceuthophilus*) were placed sister to the Macropathinae and Aemodogryllinae, and the 31 Macropathinae representatives are monophyletic with respect to the three other subfamilies sampled (figure 4). We inferred the common ancestor of the sampled Rhaphidophoridae existed during the Jurassic period and the common ancestor of the sampled Macropathinae dates to the boundary of the Jurassic and Cretaceous (approx. 142 Ma; figure 4). If these estimates are broadly correct, then the Rhaphidophoridae subfamilies may have already diverged before Pangaea broke apart. The majority of the New Zealand endemic genera shared common ancestors during the Eocene (55–34 Ma), suggesting that much of the phylogenetic diversity currently in the New Zealand region had an origin before the Oligocene. We sampled from five subantarctic islands including four that have endemic genera of Rhaphidophoridae (table 2). In four cases, the island taxa are sister to lineages sampled from mainland New Zealand, the exception being *Novoplectron serratum* (from Chatham Islands) sister to *Ischyroplectron isolatum* (from Bounty Island). For all subantarctic lineages, age estimates of their most recent common ancestor are significantly older than the land surfaces of all five islands (table 2).

**Table 2.** The age of outlying New Zealand islands and the inferred age of the cave wētā endemic to the islands. Time since most recent common ancestor between mainland and offshore species inferred from dating whole mitochondrial genome phylogenetic tree and sampling 16 genera endemic to New Zealand. Range for date estimate is 95% highest posterior density (HPD).

rhaphidophorid island endemic	island	age of land above the sea and/or free of ice	reference for island age	time since most recent common ancestor from molecular clock
*Novoplectron serratum*	Chatham Islands Rēkohu	uplift 2–3 Ma 3–4 Ma	[29,30]	39.7 Ma (28.1–51.7)
*Talitropsis crassicruris* & *megatibia*	Chatham Islands Rēkohu	uplift 2–3 Ma 3–4 Ma	[29,30]	9.8 Ma (6–14.6)
*Dendroplectron aucklandense*	Auckland Islands Motu Maha	volcano 16 Ma Ice-covered 18 ka	[28,61]	57.7 Ma (45.2–71.4)
*Notoplectron campbellense*	Campbell Islands Motu Ihupuku	volcano 11–6 Ma ice covered 18 ka	[28,61]	26.6 Ma (17.8–36.1)
*Ischyroplectron isolatum*	Bounty Island Moutere Hauriri	less than 20 Ma	[28,62]	39.7 Ma (28.1–51.7)
*Insulanoplectron spinosum*	Snares Island Tini Heke	less than 20 Ma	[28,62]	44.7 Ma (32–58)

## 4. Discussion

The evolutionary relationships among the New Zealand rhaphidophorid taxa inferred using mitochondrial and nuclear DNA sequence data were broadly concordant and consistent with recent systematic changes including resurrection of the genus *Miotopus* [32], and transfer of *Pharmacus brewsterense* to the genus *Notoplectron* [27]. However, our phylogenetic inference suggests a close relationship between the two *Notoplectron* species sampled and the monotypic *Maotoweta virescens* Johns & Cook 2014, consistent with their synonymy. By contrast, two undescribed New Zealand taxa included in this analysis represent novel lineages consistent with the establishment of two new genera.

The Southern Hemisphere cave wētā form a clade of Macropathinae that are sister to the Aemodogryllinae of Asia. If the fossil calibrations constraining our molecular clock analysis are reliable, then the common ancestor of the Northern Hemisphere Rhaphidophoridae was older than previously estimated. The uncertainties inherent in fossil calibrations are integrated into molecular dating using the Bayesian framework [63], but clock analyses rely on the availability of fossils that are phylogenetically well defined. Our analysis depends on a fossil lineage that, although supported by a large number of well-dated fossil species and genera, is not directly part of the focal family (Rhaphidophoridae), so that rate variation among groups has the potential to lead to unrealistic models. The most recent common ancestor of Ceuthophilinae (from North America) and Troglophilinae (from Europe) was estimated to be about 70 Ma using geological calibrations [24], but our fossil-calibrated analysis suggests 146 Ma (95% HPD 100–192) for this common ancestor. Similarly, our estimate for the common ancestor of the Macropathinae and Aemodogryllinae at 163 Ma is about 45 Myr earlier than previously thought (95% HPD 142–230.2 Ma). Because the 95% HPDs are wide, if the maximum estimates from geological calibrations are compared with minimum estimates from fossil calibrations, then the differences are just 5–10 Myr [24]. Our fossil-calibrated molecular clock analysis yielded divergence dates more compatible with studies of other Orthoptera that incorporate fossils into their clock calibrations (e.g. [43,64,65]), but not analyses based on geology or substitution rates derived from geological events (e.g. [26]).

As expected, mtDNA and nuclear phylogenetic evidence suggests that the Australian and New Zealand Macropathinae are not reciprocally monophyletic clades [24,26]. Notably, the Tasmanian species *Parvotettix domesticus* is sister to the rest of the Macropathinae diversity we sampled, and they share a common ancestor about 140 Ma (95% HPD 104.8–192.9 Ma). Two species from South America nest within the phylogenetic diversity endemic to New Zealand, but they shared a common ancestor with New Zealand taxa at about the boundary between the Cretaceous and Palaeogene. We note that the 95% highest posterior density interval (56.5–92.1 Ma) for this common ancestor spans the period when Zealandia was rifted from Gondwana [66].

Our phylogenetic inferences suggest that many lineages within the extant Macropathinae may have diverged prior to the break-up of the Southern continents; however, long-distance dispersal has nevertheless been an important biogeographic process. This is evident in the existence of seven species endemic to outlying islands of New Zealand that required trans-oceanic dispersal for colonization, and previous studies have inferred a role for dispersal in rhaphidophorid's distribution [24,67]. In New Zealand, many forest Macropathinae make use of holes in living and dead wood for refuge during the day [68] and some lay eggs into wood, providing opportunity for accidental transportation during floods [69]. The ability of Macropathinae to colonize isolated oceanic islands in short time frames (less than 20 000 years for subantarctic islands) and sometimes with multiple independent arrivals (e.g. endemic monotypic *Novoplectron serratum*, and endemic species of *Talitropsis* on Chatham Islands), suggests that dispersal is a significant factor shaping their diversity and distribution across the Southern Hemisphere.

Four of the New Zealand subantarctic islands have an endemic species that belongs to its own unique genus, suggesting a close relative has not been identified anywhere. If our fossil calibration is correctly constraining our molecular clock analysis, then the most recent common ancestors of the island-endemics and the mainland taxa sampled existed many millions of years before the islands were inhabitable. The finding of ‘old lineages' whose divergence times pre-date the island emergence is not uncommon; in the Chatham Islands alone 20% of lineages studied pre-date its emergence [21]. Divergence does not date from colonization, so for at least five of the Macropathinae taxa examined here ancestors must have existed on mainland New Zealand or elsewhere until quite recently (Last Glacial Maximum (LGM) or 2 Ma) but have not been sampled. Failure to sample could be the result of extinction or because taxa have yet to be discovered, and this might be because they have remained cryptic in local environments, or exist in more distant landscapes. Since 2018, 14 new species of Rhaphidophoridae endemic to New Zealand have been described, so failure to discover sister taxa is possible. Although these new species exist within known lineages, two new genera on long phylogenetic branches have also been revealed by our data. Despite our sampling encompassing all genera known from the New Zealand region and two previously not known, we still have five island taxa with common ancestors much older than their island homes, and therefore it seems likely that extinction has played an important role in the current patterns of diversity illustrated by this group.

## Data Availability

All data used in this study are publicly available; the DNA sequences are on GenBank (accession nos. OR520204–OR530267; OR551709–OR551736) and alignments are available at the Dryad Digital Repository [52]: https://doi.org/10.5061/dryad.nk98sf7z3. The data are provided in electronic supplementary material [70].
